# A Novel Approach to Medical Student Peer-assisted Learning Through Case-based Simulations

**DOI:** 10.5811/westjem.2017.10.35319

**Published:** 2017-12-18

**Authors:** Joshua Jauregui, Steven Bright, Jared Strote, Jamie Shandro

**Affiliations:** University of Washington School of Medicine, Department of Emergency Medicine, Seattle, Washington

## Abstract

**Introduction:**

Peer-assisted learning (PAL) is the development of new knowledge and skills through active learning support from peers. Benefits of PAL include introduction of teaching skills for students, creation of a safe learning environment, and efficient use of faculty time. We present a novel approach to PAL in an emergency medicine (EM) clerkship curriculum using an inexpensive, tablet-based app for students to cooperatively present and perform low-fidelity, case-based simulations that promotes accountability for student learning, fosters teaching skills, and economizes faculty presence.

**Methods:**

We developed five clinical cases in the style of EM oral boards. Fourth-year medical students were each assigned a unique case one week in advance. Students also received an instructional document and a video example detailing how to lead a case. During the 90-minute session, students were placed in small groups of 3–5 students and rotated between facilitating their assigned cases and participating as a team for the cases presented by their fellow students. Cases were supplemented with a half-mannequin that can be intubated, airway supplies, and a tablet-based app (SimMon, $22.99) to remotely display and update vital signs. One faculty member rotated among groups to provide additional assistance and clarification. Three EM faculty members iteratively developed a survey, based on the literature and pilot tested it with fourth-year medical students, to evaluate the course.

**Results:**

135 medical students completed the course and course evaluation survey. Learner satisfaction was high with an overall score of 4.6 on a 5-point Likert scale. In written comments, students reported that small groups with minimal faculty involvement provided a safe learning environment and a unique opportunity to lead a group of peers. They felt that PAL was more effective than traditional simulations for learning. Faculty reported that students remained engaged and required minimal oversight.

**Conclusion:**

Unlike other simulations, our combination of brief, student-assisted cases using low-fidelity simulation provides a cost-, resource- and time-effective way to implement a medical student clerkship educational experience.

## BACKGROUND

Peer-assisted learning (PAL) is the development of new knowledge and skills through active learning support from peers. It specifically involves individuals who are peers, not professional teachers, and who are also learning themselves through teaching. 1,2 Furthermore, it is not simply a group activity or cooperative problem-solving in which peers are not directly teaching or assessing. 3 In short, it is medical students teaching medical students.

PAL is primarily founded upon the theories of social constructivism and cognitive congruence. 4, 5 Social constructivism is characterized by the process of learning in a group setting towards the development of shared meaning created by the collaborative interaction itself. 6 PAL is informed by this theory in that students learn from peers in a social setting towards a common goal of understanding. 5 The more commonly cited theory underpinning peer-assisted learning initiatives is cognitive congruence. 1,5,7 Cognitive congruence focuses on the relative gap in knowledge between a student and an instructor and states that the relatively smaller gap between student teachers and student learners allows for the enhancement of communication of facts and understanding. 8 Essentially, students are more apt to explain content in relatable ways to each other.

Beyond the theories that form the basis of PAL, PAL may be effective for a number of other reasons. Student learners are often more engaged in active learning and at ease to ask questions and clarify understanding with fellow students, while student teachers may deepen their understanding and improved retention in a topic they prepared for and delivered. Furthermore, an understanding of the learning process and the ability to teach is an important skill for competent resident physicians to possess, for teaching junior learners and for teaching patients about their conditions. PAL allows students to participate in teaching in a safe learning environment in anticipation of becoming teachers themselves when they graduate to residency. 9, 5, 10 Also, PAL inherently encourages students to take responsibility for their own learning and develop critical lifelong learning skills. From the educator’s standpoint, faculty time is a limited resource that can be the rate-limiting step in providing interactive learning experiences for medical students. PAL offers an active learning education approach that is also an efficient use of faculty time.

Studies evaluating the effectiveness of PAL have demonstrated a positive effect on student teachers’ performance, concluding that students are more likely to retain concepts they teach and that they often spend more time preparing for topics they are expected to teach. 11 In addition, research on the effect of student learners’ performance has demonstrated that PAL increased improvement in objective structured clinical examination (OSCE) and test scores. 5 The instances in which PAL had no effect or negatively impacted performance in the literature were largely due to complex content beyond the skills of student teachers to deliver. 12

There is a paucity of research on PAL in emergency medicine (EM). One recent article on the effective use of peer teaching for medical students demonstrated that students’ response to peer teaching was positive and they learned equally well. 13 This example may under-represent the benefits of PAL, as they used high-fidelity simulations, which demand a higher preparation time and cognitive load for student learners. Additionally, implementation of high-fidelity PAL simulations may also be less feasible and less cost effective to replicate.

Given the many positive attributes of PAL, we present a novel PAL curriculum in an EM clerkship curriculum using an inexpensive, tablet-based application for students to cooperatively present and perform low-fidelity, case-based simulations.

## OBJECTIVES

Our goal was to develop and implement a case-based PAL session that promotes accountability for student learning, fosters teaching skills, and economizes faculty presence.

## CURRICULAR DESIGN

This innovation was a part of a required month-long EM clerkship for fourth-year medical students at two academic sites. Students in the clerkship are expected to learn primarily through direct patient experience and bedside teaching, supplemented with virtual lectures, directed readings, labs and simulation experiences. They are required to attend two lab days that involve clinical workshops and simulations. The first lab day is conducted during their first week of the clerkship and includes high-fidelity simulations focusing on Acute Cardiac Life Support cases. The second lab day is conducted during the second week of the clerkship and involves the innovative PAL cases.

We developed five clinical cases representing a range of acute conditions seen in EM in the style of EM oral boards (Table 1). We chose this style to provide a consistent and familiar case structure for faculty supervisors and to allow each student teacher preparing for their own case to become familiar with the structure of the cases that their fellow students would present. One week in advance of the session, students were assigned one of the five unique cases and were given a colored folder containing their case and a three-page document explaining the overall session and expectations. They also received a link to a 13-minute video tutorial on how to use the software they would be using to teach, and a video demonstrating an example case. Expected preparation time for students was less than one hour.

The learning objectives are outlined for the student teacher to review, and each case file starts with an overview of the patient’s clinical course. The case starts in a typical oral boards format with the student teacher verbalizing the chief complaint, vital signs, and general appearance. The student teacher then responds to any questions or actions from the student learners in their group, and the student teacher acts as facilitator, nurse, and consultant as needed. There are prompts for the student teacher to help guide the student learners when the team should perform critical actions in each case. For example, in a patient with sepsis for whom the students are expected to order a lactate with labs, the case has the prompt: “If the team fails to get a lactate then ask, ‘Doctor, you mentioned sepsis in your differential. Aren’t there guidelines on what labs we need if we are suspecting sepsis?’” There are also prompts for timing, and for concluding the case by 10–12 minutes to give time to debrief and teach. At the conclusion of each case, there is a guide for the student teacher to conduct a debriefing with their group as well as to discuss some brief outlined learning points specific to the case.

An iPad-based (Apple Inc.©) application, SimMon (Castle+Andersen ApS ©, $22.99), served as a low-fidelity, simulated monitor. The student teacher had access to one iPad, which served as the remote, and it was linked via Bluetooth® to a second iPad serving as the display monitor for the student participants. Each student watched a video tutorial on how to use the application. One device pairs with the other; one serves as the remote while the other as the display. Manipulable variables include heart rate, cardiac rhythm, respiratory rate, oxygen saturation and blood pressure. They are changed simply by moving one’s finger up or down on the remote control screen. There is also a timer to help students track time for their case. Each student watched a short video tutorial demonstrating an example of how to lead a case while using the application.

Each PAL session lasted 90 minutes. Students were placed in small groups of 4–5 students sorted into groups by the same folder color that contained their teaching case to ensure that each group would have a collection of unique cases. Students rotated between facilitating their assigned cases and participating as a team for the cases presented by their fellow students. Cases were supplemented with a half-mannequin that could be intubated, airway supplies and the tablet-based application described above to remotely display and update vital signs. One faculty member rotated among two or three groups to provide additional assistance and clarification. The faculty member also received a facilitator guide to outline the session objectives, and was directed to allow students to primarily guide the teaching session so as not to undermine the actualization and benefits of PAL while still proving content expertise and ensuring educational quality.

As one of the benefits of PAL is a safe learning environment, we deliberately decided not to have a summative evaluative assessment of this session. The sessions are a mandatory part of the clerkship, and the PAL format fosters an intrinsic accountability to fellow students such that students had an innate motivation to prepare for their roles as student teachers and learners.

This curricular evaluation was determined to be exempt from review by the University of Washington Human Subjects Division. We learned several lessons during initial implementation that helped us improve these sessions. We found that for a 90-minute educational session, it worked best to allocate groups of four students with 20 minutes for each case and debrief. We also found that a single faculty or resident supervisor could easily monitor and assist with three groups, or 12 fourth-year medical students. We added the color-coded folders to keep groups organized and ensure unique case representation in groups. Other updates that occurred during the implementation period in response to student and faculty insights included small medication adjustments, the addition of more electrocardiograms and radiology images, and minor clarifications in the wording in some guides.

## IMPACT

Three EM faculty members with advanced training in medical education research iteratively developed a survey to assess the course. Validity evidence of this survey assessment was built upon Messick’s validity framework. 14, 15 The survey was based on the literature and the theories underpinning PAL. It focused on three domains: content of the simulations, the students’ experience guiding their simulation, and their experience learning from their peers. It was pilot tested for response process with four fourth-year medical students prior to survey administration, and their feedback was incorporated into the final version of the survey. Overall internal consistency of the survey was excellent with a Cronbach’s alpha of 0.98, as was the internal consistency of each domain, with a Cronbach’s alpha of 0.92, 0.94, and 0.95 respectively for content of the simulations, the students’ experience guiding their simulation, and their experience learning with their peers.

A total of 160 students completed the course over an 11-month period between June 2016 and April 2017. One hundred thirty-five students completed the course evaluation survey for a response rate of 84%. Learner satisfaction was high with an overall score of 4.6 (standard deviation of 0.7) on a 5-point Likert scale with 88% of respondents either agreeing or strongly agreeing that their overall learning with peers in this format was a positive experience (Table 2). Students felt that the cases covered concepts appropriate for their level of training and that running a simulation for their peers was better for retaining new concepts than being a participant. They also responded that running the simulation did not require too much additional work or time. In written comments, students reported that small groups with minimal faculty involvement provided a safe learning environment and a unique opportunity to lead a group of their peers. In written comments, faculty reported that students remained engaged and required minimal oversight.

A limitation of our curricular evaluation is that we only measured Level 1 Kirkpatrick outcomes. 16 In future work, we plan to measure higher level outcomes, such as translation into practice with simulated assessments. Other future directions for this project include a pilot of distance participation in PAL simulations with students rotating concurrently in remote sites on their EM clerkships, using teleconferencing technology. We would also like to develop a non-summative knowledge assessment to evaluate if students are meeting the knowledge-based objectives of this session.

## CONCLUSION

In summary, our EM clerkship peer-assisted learning case sessions were well received by both students and faculty and were feasible to implement. We anticipate that this curricular innovation could be readily implemented at other institutions with a small investment in tablets and the simulation application, or with other low-fidelity simulation options. We found that our PAL cases using low-fidelity simulation provide a cost-, resource- and time-effective way to implement an active medical student clerkship educational experience.

## Supplementary Information



## Figures and Tables

**Table 1:** Clinical cases and key directed teaching points for peer-assisted learning cases in an emergency medicine clerkship

Clinical case	Key teaching points
Supraventricular tachycardia, 37-year-old female	Review pathology and findings with Wolff-Parkinson-White (WPW) syndrome. Review the ACLS algorithm for tachycardia
Subdural hemorrhage, 48-year-old male	Briefly review the differential of altered mental status Discuss the importance of maintaining a broad differential for patients with altered mental status.
Aortic dissection, 64-year-old female	Review classification of aortic dissection. Review initial treatment for aortic dissection. Discuss components of an effective consult.
Ectopic pregnancy, 31-year-old female	Review risk factors for ectopic pregnancy. Consider obtaining a pregnancy test for every woman of reproductive age in the ED. Review indications for Rho immune globulin (RhoGam).
Sepsis, 82-year-old female	Review definitions of SIRS, sepsis, severe sepsis and septic shock. Early recognition and treatment of patients with sepsis improves morbidity and mortality. Obtain a lactate early. If the lactate is elevated, repeat lactate after fluid resuscitation.

*ED*, emergency department.

**Table 2:** Peer-assisted learning survey results.

Statement posed to subjects (n=135)	Mean Score	Standard deviation	% Agree or Strongly Agree
The peer-guided simulations covered concepts that were appropriate for my knowledge base and experience	4.8	0.4	99%
Participating in the peer-assisted simulations helps me feel better prepared for my exams and clinical experience	4.5	0.7	91%
Participating in the peer-guided simulations will help me retain new concepts and skills better than faculty facilitated simulations	4.4	0.8	85%
Running a peer-guided simulation did not require too much additional work or time outside of this rotation	4.5	0.7	93%
Running a simulation for my peers will help me retain new concepts and skills better than just participating in a simulation	4.5	0.8	86%
Running a simulation makes me more likely to engage in teaching activities in the future	4.1	0.9	69%
My fellow students were well prepared to run the peer-guided simulations	4.3	0.8	84%
Overall, learning with my peers in this format was a positive experience	4.6	0.7	88%
I found the peer-guided simulations more interactive than previously experienced faculty facilitated simulations	4.2	1.0	74%

Ratings on 5-point Likert scale

1=Strongly disagree, 2=Disagree, 3=Neutral, 4=Agree, 5=Strongly agree
