# Supplementary material for: A Novel Approach to Medical Student Peer-assisted Learning Through Case-based Simulations
Source: West J Emerg Med. 2017 Dec 18;19(1):193–7. doi: 10.5811/westjem.2017.10.35319 (PMC5785193; doi:10.5811/westjem.2017.10.35319)
Supplement: Supplementary file 1 [file wjem-19-193-s001.docx]

**Appendix 1.** Survey given to medical students after participation in peer-assisted learning session

**Peer-guided Simulation Feedback**

These questions were developed to better understand your experience participating in the peer- guided simulations

**The following questions pertain to the content of the simulations:**

1. The peer-guided simulations covered concepts that were appropriate for my knowledge base and experience.
   1. Strongly Agree
   2. Agree
   3. Neutral
   4. Disagree
   5. Strongly Disagree
   6. Comments _____________________________

2. Participating in the peer-guided simulations helps me feel better prepared for my exams and clinical experience.

- 1. Strongly Agree
  2. Agree
  3. Neutral
  4. Disagree
  5. Strongly Disagree
  6. Comments _____________________________

3. Participating in the peer-guided simulations will help me retain new concepts and skills better than faculty facilitated simulations.

1. Strongly Agree
2. Agree
3. Neutral
4. Disagree
5. Strongly Disagree
6. Comments _____________________________

**The following questions pertain to your experience guiding your simulation:**

1. Running a peer-guided simulation did not require too much additional work or time outside of this rotation.
2. Strongly Agree
3. Agree
4. Neutral
5. Disagree
6. Strongly Disagree
7. Comments _____________________________
8. Running a simulation for my peers will help me retain new concepts and skills better than just participating in a simulation.
   1. Strongly Agree
   2. Agree
   3. Neutral
   4. Disagree
   5. Strongly Disagree
   6. Comments _____________________________
9. Running a simulation makes me more likely to engage in teaching activities in the future
   1. Strongly Agree
   2. Agree
   3. Neutral
   4. Disagree
   5. Strongly Disagree
   6. Comments _____________________________

**The following questions pertain to your experience learning with your peers**

1. My fellow students were well prepared to run the peer-guided simulations.
2. Strongly Agree
3. Agree
4. Neutral
5. Disagree
6. Strongly Disagree
7. Comments _____________________________

2. Overall, learning with my peers in this format was a positive experience

1. Strongly Agree
2. Agree
3. Neutral
4. Disagree
5. Strongly Disagree
6. Comments _____________________________

3. I found the peer-guided simulations more interactive than previously experienced faculty facilitated simulations.

1. Strongly Agree
2. Agree
3. Neutral
4. Disagree
5. Strongly Disagree
6. Comments _____________________________

Short answer: What would you suggest we change or do differently next time?
